# Personalization matters: the effect of sex in multivitamin-multimineral-based cancer prevention

**DOI:** 10.1007/s11357-023-00882-7

**Published:** 2023-08-10

**Authors:** Julij Šelb, Filip Cvetko, Leon Deutsch, Leon Bedrač, Enej Kuščer, Andrea Britta Maier

**Affiliations:** 1 The NU B.V., J.H. Oortweg 21, 2333CH Leiden, The Netherlands; 2 University Clinic of Respiratory and Allergic Diseases Golnik, Golnik 36, 4204 Golnik, Slovenia; 3 Department of Human Movement Sciences, @AgeAmsterdam Amsterdam Movement Sciences Vrije Universiteit Amsterdam, Amsterdam, The Netherlands; 4 Healthy Longevity Translational Research Program, Yong Loo Lin School of Medicine, National University of Singapore, Singapore, Republic of Singapore; 5 Centre for Healthy Longevity, @AgeSingapore, National University Health System, Singapore, Republic of Singapore

**Keywords:** Primary cancer prevention, Dietary supplements, Vitamins/minerals, Sex/gender, Meta-analysis

## Abstract

The prevention of non-communicable diseases like cancer contributes to healthy aging. Dietary supplements might support such prevention; their effect likely depends on the personal characteristics of the individuals receiving them. To evaluate the influence of sex on reducing cancer incidence with multivitamin-multimineral (MVM) supplementation, sex-specific results of the efficacy of MVM supplementation for cancer prevention were collected and meta-analyzed (using fixed effect (FE) and random effect (RE) models). Three trials included in the “US Preventive Services Task Force Recommendation Statement Report regarding Vitamin, Mineral, and Multivitamin Supplementation to Prevent Cardiovascular Disease and Cancer” were used, namely, COSMOS, SU.VI.MAX, and PHS2. A total of 28,558 men and 20,542 women were included. Multivitamin-multimineral supplementation significantly reduced cancer incidence in the entire population (HR 0.93 [95% CI, 0.88–0.99], FE and RE); sex-specific meta-analysis showed beneficial effects of supplementation in men (HR 0.91 [95% CI, 0.85–0.97] (FE)/0.88 [95% CI, 0.77–1.01] (RE)); however, there was no effect in women (HR 1.00 [95% CI, 0.88–1.14], FR and RE); (*P*_difference_ = 0.17). Sex could influence the effect of MVM supplementation in reducing cancer incidence, with supplementation being effective only in male individuals. These results might be informative for future research and public health policy makers.

**Supplementary Information:**

The online version contains supplementary material available at 10.1007/s11357-023-00882-7.

## Introduction

The prevention of non-communicable diseases like cancer contributes to healthy aging. The use of dietary supplements represents a possible venue for such prevention. Traditionally, clinical studies testing the efficacy of dietary supplements in cancer prevention pursued a “one size fits all” approach, not adjusting the supplementation to the biological characteristics of individuals involved in trials. However, multiple recent studies refuted such an approach by showing that sub-groups of individuals who share a specific biological property may benefit from supplementation-based interventions, while for individuals lacking such a property, supplementation can be ineffective or even harmful. Sub-groups defining biological properties (effect modifiers) range from BMI [1, 2] and sex [3] on one side to the genetic makeup of an individual on another [4].

A meta-analysis of three primary preventive trials, namely, COcoa Supplement and Multivitamin Outcomes Study (COSMOS) [5], Supplementation en Vitamines et Mineraux Antioxydants (SU.VI.MAX) [6], and Physicians' Health Study II (PHS2) [7], published as part of the US Preventive Services Task Force Recommendation Statement Report (USPSTFRSR) regarding vitamin, mineral, and multivitamin supplementation to prevent cardiovascular disease and cancer, detected a statistically significant, albeit small (OR 0.93 [95% CI, 0.87–0.99]), decrease in cancer incidence in the general adult population with multivitamin-multimineral (MVM) supplementation [8]. In all three trials, sex might have influenced the outcomes. PHS2 included only male participants [7]; in SU.VI.MAX [6], supplementation was effective only in men while being ineffective in women (*P*_interaction_ = 0.004); and in COSMOS, the effect of MVM supplementation was non-significantly better in men than in women [5].

Multiple hypotheses for the assumed differences in the effect of MVM supplementation between men and women exist pertaining to biological as well as socio-cultural distinctions between the two [9]. Men are likely to have less healthy eating patterns than women (i.e., eating less fruits, vegetables, and fibers) [10, 11]; this, together with differences in absorption capacity for various micronutrients [12], might result in sub-optimal nutritional status of the male population (as possibly observed in the SU.VI.MAX trial) [6]. Moreover, biological differences between men and women could lead to different metabolic requirements for a specific nutrient/micronutrient [9].

To further explore the role of sex in MVM supplementation-based cancer prevention, a sex-stratified meta-analysis of the three trials included in the USPSTFRSR was constructed and tested sex as a possible effect modifier of the efficacy of the intervention.

## Methods

### Characteristics of included studies

The meta-analysis included all three cancer primary-preventive trials that were used in the USPSTFRSR MVM meta-analysis, namely, COSMOS [5], SU.VI.MAX [6], and PHS2 [7]. All three included trials were large (*n* > 10,000 individuals), well-powered, randomized, double-blind, placebo-controlled trials with a primary endpoint of primary cancer prevention. COSMOS and SU.VI.MAX were conducted on a general adult population, while PHS2 was done on a male-only adult population. Moreover, while COSMOS and PHS2 were done in the USA, the SU.VI.MAX trial was conducted in France. The composition of the MVM preparation used in COSMOS/PHS2 and SU.VI.MAX differed; the MVM preparations used in COSMOS/PHS2 consisted of about 30 different supplements (the dose of each supplement was roughly similar between the two trials), while the SU.VI.MAX formula consisted of 5 supplements—3 antioxidants and 2 minerals. All the supplements present in SU.VI.MAX were also present in COSMOS/PHS2; however, the doses given in SU.VI.MAX were higher than COSMOS/PHS2 doses. The age distribution of participants included in studies varied; while COSMOS and PHS2 consisted of mainly older individuals (the mean age in COSMOS was 72 years and the mean age in PHS2 was 64 years), the mean age of females/males in SU.VI.MAX was 47/51 years.

### Statistical analysis

Estimates of the effect sizes of MVM supplementation on cancer incidence, expressed in terms of hazard/risk ratios (HR/RR) with a 95% confidence interval (CI), for all three trials were collected and meta-analyzed (Table 1). A general and a sex-specific meta-analysis of the results were performed with fixed-effect (FE) and random-effect (RE) models. For the FE model, the meta-analyzed estimation of the effect size was derived using weighted estimations of the effect sizes of the three trials with inverse-variance weights. The “restricted maximum likelihood estimator” was used as the estimator of the amount of heterogeneity in the RE model. Furthermore, a meta-regression analysis (FE/RE) to test for the difference between sex and the effect of MVM supplementation was conducted. All analyses were done using R’s metafor package [13].

**Table 1:** Effect of supplementation and the number of participants in each of the 3 RCTs that specifically focused on MVM supplementation for cancer prevention

	HR/RR [95% CI] (*n*)—male	HR/RR [95% CI] (*n*)—female	HR/RR [95% CI] (*n*)—male + female
COSMOS	0.94 [0.78–1.13] (*n* = 8776)	0.98 [0.84–1.15] (*n* = 12,666)	0.97 [0.86–1.09] (*n* = 21,442)
SU.VI.MAX	0.69 [0.53–0.91] (*n* = 5141)	1.04 [0.85–1.29] (*n* = 7876)	0.90 [0.76–1.06] (*n* = 13,017)
PHS2	0.92 [0.86–0.998] (*n* = 14,641)	/	/

## Results

The analysis included a total of 49,100 individuals (flowchart – supplementary material). With regard to the number of participants, the biggest contributor was COSMOS (21,442 individuals; 8776 men/12,666 women), followed by PHS2 (14,641 individuals; 14,641 men/0 women) and SU.VI.MAX (13,017 individuals; 5141 men/7876 women). The sex-stratified analysis thus consisted of 28,558 men and 20,542 women (Table 1).

**Fig. 1:**
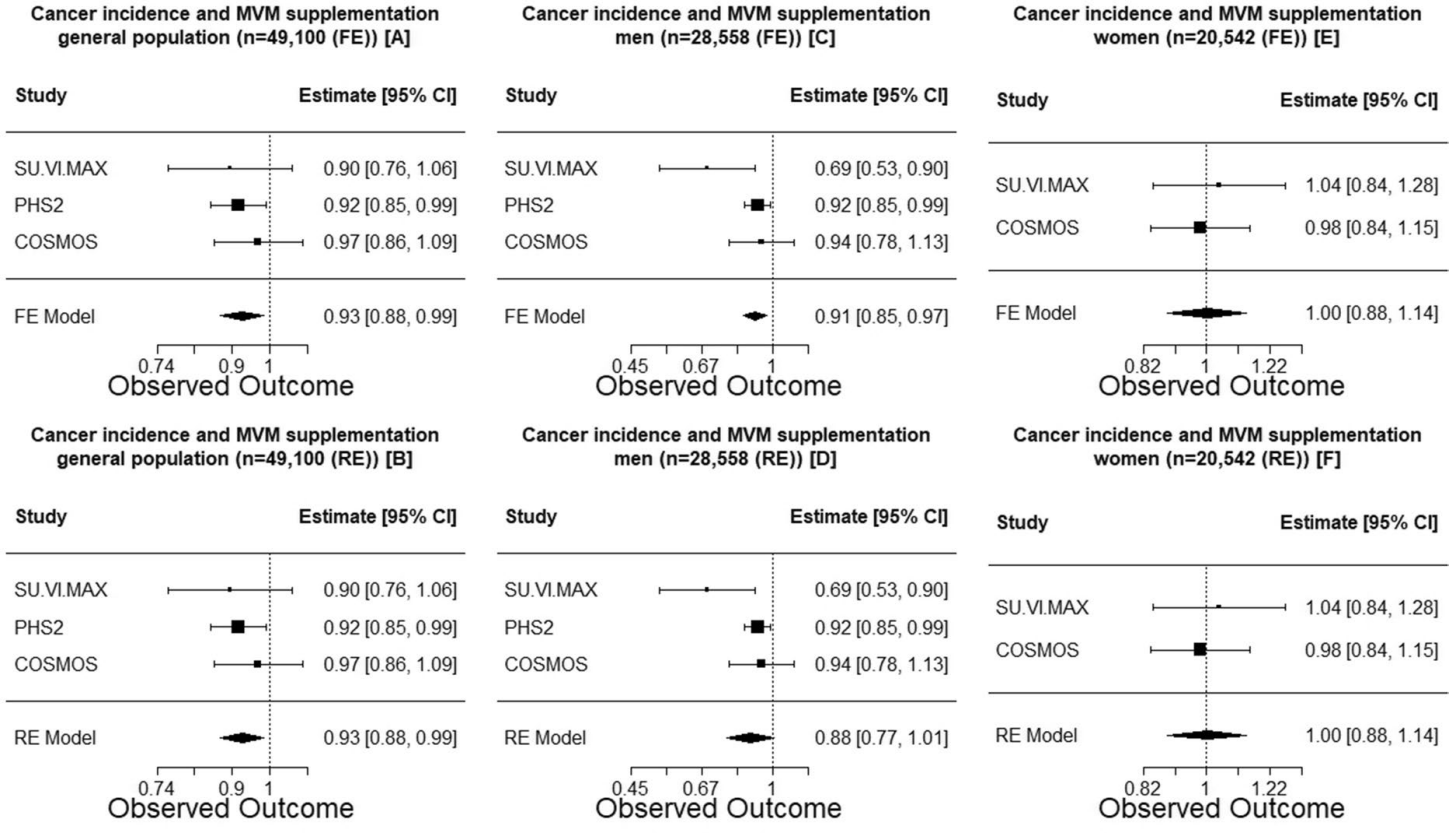
Sex specific meta-analysis of the 3 RCTs that specifically focused on MVM supplementation for cancer prevention. FE, fixed effects model; RE, random effects model

Multivitamin-multimineral supplementation significantly reduced cancer incidence in the entire population (HR 0.93 [95% CI, 0.88–0.99], FE and RE; Fig. 1A, B); sex-specific meta-analysis indicated a beneficial effect of supplementation in men (HR 0.91 [95% CI, 0.85–0.97] (FE)/0.88 [95% CI, 0.77–1.01] (RE); Fig. 1C, D); however, there was no effect in women (HR 1.00 [95% CI, 0.88–1.14], FR and RE; Fig. 1E, F). The results of the effects of MVM supplementation according to sex in the meta-regression (FE/RE) analysis were similar (Table 2); in men, the HR of supplementation on cancer incidence was 0.91 (95% CI, 0.85–0.97), while in women, it was 1.00 (95% CI, 0.88–1.14); however, the difference was not statistically significant (*P*_difference_ = 0.17).

**Table 2:** Results of the meta-regression analysis

	HR [95% CI] (RE/FE)	*P*-value	*P*-value difference
Male	0.906 (0.848–0.969)	0.004	0.17
Female	1.001 (0.883–1.135)	0.984	

## Discussion

The current study extends the results of the USPSTFRSR regarding MVM supplementation and its influence on cancer incidence [8]; the significant beneficial effect of MVM supplementation described in the report likely reflects the benefit present in male individuals since supplementation did not affect cancer incidence in women. Such sex-specific results are more informative as to who can benefit from MVM supplementation as are the “general population” results presented by the USPSTFRSR.

For the sex-stratified meta-analysis, we used the FE and RE models since debate still exists about the proper use of the two models [14]. The fixed-effects model assumes that one true effect size exists in the population; RE assumes that the true effect varies (and is normally distributed). In most instances, the results between the models did not differ. The only difference was present in the male population, where RE produced a slightly lower HR estimate with a bit wider confidence interval, likely reflecting the higher variability of the results of male individuals (HR and confidence intervals) present in the original studies (i.e., SU.VI.MAX vs. COSMOS). The meta-regression analysis, similarly, revealed a significantly lower cancer incidence with MVM supplementation in males, but the between-sex difference was not statistically significant. This could be because PHS2, the biggest contributor to the overall result in the male population, did not include women and consequently did not add to the difference.

As mentioned, few explanations for the observed sex-specific results exist; however, they are only speculative. Men likely have less healthy eating patterns than women (i.e., eating less fruits, vegetables, and fibers) [10, 11]; this, together with a difference in the absorption capacity for various micronutrients [12], might result in sub-optimal nutritional status for the male population. Compared to women, in SU.VI.MAX, men had lower baseline levels of β-carotene and vitamin-C [6] (both, β-carotene and vitamin-C, were supplemented ingredients) which both had risen in the post-supplementation period. The improved nutritional status for selected micronutrients post-supplementation might provide an explanation for the observed reduction in cancer incidence [6]. While unrelated to sex, in the COSMOS trial, the biggest trend of the efficacy of MVM supplementation (which was, however, statistically non-significant) was present in individuals that did not take prior dietary supplements (HR 0.84; 95% CI, 0.67–1.06) and in individuals that consumed less than 4 servings of fruits and vegetables per day (HR 0.89; 95% CI, 0.72–1.10) [5]. These data (SU.VI.MAX and COSMOS) might support the hypothesis that MVM supplementation corrects general sub-optimal nutritional status, which might be more prevalent in male individuals [7, 9]. It is worth noting that individuals in PHS2 (which consisted of only males), where MVM supplementation likewise reduced cancer incidence, consumed on average 4.2 servings (4.26 in the active group and 4.19 in the placebo group) of fruits and vegetables per day [7] which is in line with American Heart Association recommendations for adequate (quality) food intake (4 to 5 servings per day) [15]. Our results could therefore provide a basis for reconsidering food intake recommendations in a sex-specific manner.

Considering cancer site-specific results, the USPSTFRSR found a statistically significant benefit of MVM supplementation in reducing lung cancer incidence [8]. Since not all included studies presented site-specific as well as sex-specific results, we did not perform site-specific analysis stratified by sex. However, with regard to the reported sex-specific cancers (namely prostate and breast cancer), the MVM supplementation did not have a significant effect on any of the cancers in any of the three trials [5–7].

Our study has multiple strengths. To our knowledge, this is the first systematic (meta)analysis of the influence of sex on the prevention of cancer by MVM supplementation in the general adult population. Furthermore, the cumulative number of individuals in each sub-group is big. Moreover, in all three trials used for the meta-analysis, supplementation-based cancer prevention was the primary outcome of the trial. On the other hand, we only addressed the influence of sex on the efficacy of MVM supplementation. Populational heterogeneity and the heterogeneity of the interventions in the included trials persisted. The formulations of COSMOS [5] and PHS2 [7], where they used broad-spectrum MVMs, differed from the formulation in SU.VI.MAX which used an antioxidant-based formula [6]. However, the inclusion criteria present in the USPSTFRSR [8] were used. Since SU.VI.MAX and PHS2 represent extremes of heterogeneity in terms of MVM composition and both detected a significant positive signal of supplementation in male individuals, the underlying mechanism of action might be similar, justifying including the trials in the meta-analysis. The only included trial that did not detect a significant positive effect of MVM supplementation on cancer incidence in men was COSMOS [5]. However, this was probably due to the relatively short time frame of COSMOS (median follow-up of 3.6 years) and likely not due to the lack of effect of the intervention.

Similarly, the trials differed in age distributions; the mean age in PHS2/COSMOS was 64/72 years, while the mean age of females/males in SU.VI.MAX was 47/51 years [5–7]. Aging is associated with insufficiency of metabolism of specific micronutrients [16, 17]; this could be due to inadequate intake or deficient absorption of those micronutrients (i.e., vitamin B12, vitamin D, calcium …) [17]. Because the sex-specific cancer preventive effect did not change with age, since it was apparent in SU.VI.MAX (mean age of males was 51 years) as well as in on average 13 years older PHS2 participants and in on average ~ 20 years older COSMOS participants (the non-significant effect in COSMOS was likely due to the short time frame of COSMOS), we speculate that the metabolism of micronutrient(s) being responsible for the observed positive signal of our meta-analysis is not influenced by age. This could contrast the metabolism of “culprit” micronutrient(s) behind the preventive effect of supplementation on cognitive decline which was observed in two independent COSMOS sub-trials, namely, COSMOS-mind [18] and COSMOS-web [19]. Here, supplementation was effective in older individuals irrespective of sex. Thus, the metabolism of micronutrients driving the preventive effect on cognitive decline could be influenced by age but not by sex.

All in all, sex might influence MVM supplementation-based cancer prevention, with supplementation being effective only in male individuals. These results could be informative for future research and for healthcare policy makers. Furthermore, our results, together with other results of effect modifiers from the literature [1–4], shed light on an important aspect that has not been addressed in similar studies. There is no “one size fits all” solution in supplementation-based cancer prevention, and personalization with respect to an individual’s characteristics is essential to harvesting the full potential of preventive interventions.

### Supplementary Information

Below is the link to the electronic supplementary material.

Supplementary file1 (DOCX 27.1 KB)
