# Supplementary material for: Personalization matters: the effect of sex in multivitamin-multimineral-based cancer prevention
Source: GeroScience. 2023 Aug 10;46(1):1351–6. doi: 10.1007/s11357-023-00882-7 (PMC10828342; doi:10.1007/s11357-023-00882-7)
Supplement: Supplementary file 1 — Supplementary file1 (DOCX 27.1 KB) [file 11357_2023_882_MOESM1_ESM.docx]

Flowchart: flowchart of the studies included in the sex stratified meta-analysis with the total number of participants, the number of male and the number of female participants separately.

COSMOS

n (M/F)

21,442 (8,776/12,666)

SU.VI.MAX

n (M/F)

13,017 (5,141/7,876)

PHS2

n (M/F)

14,641 (14,641/0)

ALL

49,100

male

28,558

female

20,542
